# The Counterbalance of Skin Metabolism on Orbits and Diseases

**DOI:** 10.3390/medsci3020025

**Published:** 2015-05-12

**Authors:** Li-Fan Chuang, Chin-Kong Hsu, Hong-Nong Chou, Hung-Shih Chou, Ping-Jyun Sung, Chia-Ching Chen, Fu-Gin Chen

**Affiliations:** 1 Institute of Fisheries Science, National Taiwan University, Da’an, Taipei 106, Taiwan; E-Mail: unijohn@ntu.edu.tw; 2 Department of Dermatology, Yung-Ming Branch, Taipei City Hospital, Shilin, Taipei 111, Taiwan; E-Mail: dag27@tpech.gov.tw; 3 Graduate Institute of Physical Education, National Taiwan Sport University, Guishan, Taoyuan 333, Taiwan; E-Mail: hschou1311@gmail.com; 4 Institute of Marine Biotechnology, National Dong Hwa University, Pingtung 944, Taiwan; E-Mail: pjsung@nmmba.gov.tw; 5 Department of Money and Banking, National Chengchi University, Wenshan, Taipei 116, Taiwan; E-Mail: punpunneo@gmail.com; 6 Department of Pulmonology, Division of Internal Medicine, Yung-Ming Branch, Taipei City Hospital, Shilin, Taipei 111, Taiwan

**Keywords:** autonomic nervous system (ANS), porphyra-334 (p-344), skin, spectrum

## Abstract

Human organ functions are regulated by the nervous system. When human cells receive a message, this message is transmitted to the nervous system through a series of signal transmission processes. Skin conditions that occur after applying skin cream are closely related to signal transmission and nervous regulation. We determined the connection between signal regulation and natural rhythmic operations. The diurnal variations resulting from the earth’s rotation and indicate the relative relationships between the sympathetic nervous system and the parasympathetic nerve system. A spectrum was developed to assess neural transmission conditions by using skin signals which from Fourier transformation of the waves and established the association between the spectrum and diseases. The results could explain the relationships between the neurological illnesses and established spectrum. The objective was to promote the use of this spectrum as a new tool for conducting the nervous system tests in the future.

## 1. Introduction

### 1.1. The relationship between Water and Skin

Because approximately 99% of the molecules in the human body are water molecules [1], everything that changes in water affects the human body. The skin is an organ that covers the outer layer of the human body; therefore, observing skin reactions that are influenced by signals is easy. The top layer of the skin, the stratum corneum (SC), features a biosensor function [2]. A change in the moisture level of the SC is a critical factor that influences message transmission [3]. Therefore, stratum corneum hydration (SCH) can be altered by applying cream to the skin to induce osmosis and lower skin diffusion resistance [4]. Several studies have proposed that applying cream to the skin affects the expression of genes relevant to skin barrier homeostasis [5]. This phenomenon illustrates the change caused by incoming messages.

### 1.2. Skin and the Nervous System

Few studies have examined the correlation between neural excitation and skin conditions. Several studies have indicated that patients diagnosed with atopic dermatitis have dry skin in the autonomic dysfunction [6]. Furthermore, the sympathetic nervous system (SNS) regulates body temperature by adjusting the skin blood flow volume [7], thereby indirectly affecting the skin surface temperature [8]. Theories applied in traditional Chinese medicine indicate that acupuncture points and the nervous system (NS) are correlated [9]. In addition, the upper skin layer, which has a depth of approximately 0.25–0.40 mm, uptakes external oxygen for respiratory use [10]. The skin surface pH (SSpH) value affects gas solubility and is consequently an indicator of NS performance in skin metabolism.

### 1.3. Regularity Reaction of the Autonomic Nervous System

We infer that the model of the interaction between hydration and the autonomic nervous system (ANS) is related to fixed orbital patterns observed in the natural environment. For example, in the seventeenth century, Kepler suggested that the affinity of water with the moon influences bodily fluids that affect humans [11]. Chakraborty and Ghosh determined that the level of ANS excitation and the cycle of cardiovascular system activity are correlated with the varying phases of the moon [12,13]. In addition, the menstrual cycle, hospital admissions, and acute pathologies are considered to be related to the lunar cycle [11,14]. These phenomena are believed to be influenced by the electromagnetic fields of the earth and changes in lunar gravitational force [15]. Although there is no exact evidence indicating that the ANS is associated with gravity, the external environment may indirectly affect the human body through the effects of water.

### 1.4. Porphyra-334 and the Nervous System

Porphyra-334 (p-334), a nitrogenous compound, is used to enhance ANS performance and facilitate observing abnormality through measured skin values. The compound p-334, a mycosporine-like amino acid and a common constituent of algae and aquatic organisms [16], has been observed in high concentrations in certain algae, particularly *Porphyra* spp. and *Bangia atropurpurea* [17,18]. This compound has been proved to be an activator of cell proliferation [19] and an antioxidant [20]. Moreover, it protects against ultraviolet light [21] and exploration of its potential use in the cosmetics industry as a sunscreen agent is ongoing [22]. In addition, p-334 can absorb light energy, and more than 90% of this excitation energy can be transferred to heat, which is released to surrounding molecules [23,24]; thus, p-334 can be considered an energy-converting substance [25].

### 1.5. Objectives

In this study, we determined the connection between the rhythmic changes in the ANS caused by orbits observed in the natural environment and skin cream application. P-334 was used as an energy-converting substance to enhance ANS performance and to facilitate observing abnormality through measured skin values. In addition, we developed a system similar to electrocardiography called the “signal operation diagram” which was used to standardise the diagnostic process of cardiovascular diseases and explain the recommendations [26]. This system was also used to identify the communality between diseases and the participants whose conditions could not be explained using previously established principles.

## 2. Experimental Section 

### 2.1. Test Preparations

The extraction and analysis of p-334 are described as follows. Water was added to dry *Bangia atropurpurea* (15 mL g^−1^), which was extracted for 30 min at room temperature. The extraction was filtered using a Büchner funnel and a 0.22-µm filter bowl. High-performance liquid chromatography was used to analyze the concentration of the extraction (Hitachi L-7100 HPLC pump and Thermo UV6000LP detector) [27]. The concentration of p-334 in the extract was diluted to 0.01%. To prepare the cream, an 85% cream base was evenly mixed with 15% avocado oil (First Cosmetics Manufacture Co., Ltd., Taipei, Taiwan), and 2.5% of the diluted extraction (*v*/*v*) was added (the concentration of p-334 was lower than that in a previous study) [28]. A second batch of cream was prepared without using the extract and applied to the control group.

### 2.2. Participants and Experimental Design of the Treatment Study

Twenty-five participants were recruited for this 6-week study (age range: 25–65 year). This study was approved by the Institutional Review Board of Taipei City Hospital (Taipei, Taiwan). The participants were permitted to continue using their daily skin care products during the experimental period. The researchers applied cream to the dorsal forearms of the participants, which the right forearm was controlled. The participants were administered the cream on Saturday morning each week, and measurements were instrumentally recorded (1) before the cream was applied; (2) 20 min after application, when the forearms of the participants were washed using detergent and dried; and (3) 10 min after a second application of the cream.

### 2.3. Data Generation and Analysis

At a room temperature of 23  ± 1 °C and humidity of 52% ± 2%, the Callegari Soft Plus Skin Analyser (Callegari SpA; Parma, Italy) was employed to measure the SCH and SSpH of the dorsal forearms (at approximately 5 cm above the wrist joints and 5 cm below the elbow joints). The means of the measured data obtained from the two areas of the forearms were used as the experimental data. The SCH-SSpH values collected at all 6 weeks were plotted in diagrams. All data were analyzed using one-way analysis of variance (ANOVA) and any difference in means was considered significant at 0.05. However, there were no significant differences between left and right dorsal forearms. We used an autonomic nervous system performance diagram to compare the differences.

### 2.4. Autonomic Nervous System Performance Diagram Generation

**Figure 1:**
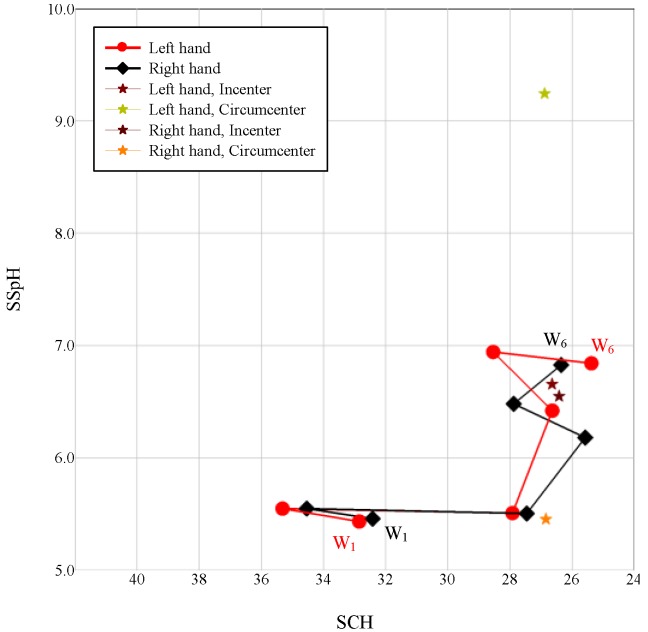
First measurement of the stratum corneum hydration (SCH)-skin surface pH (SSpH) relationship over six weeks (*N* = 25).

**Figure 2:**
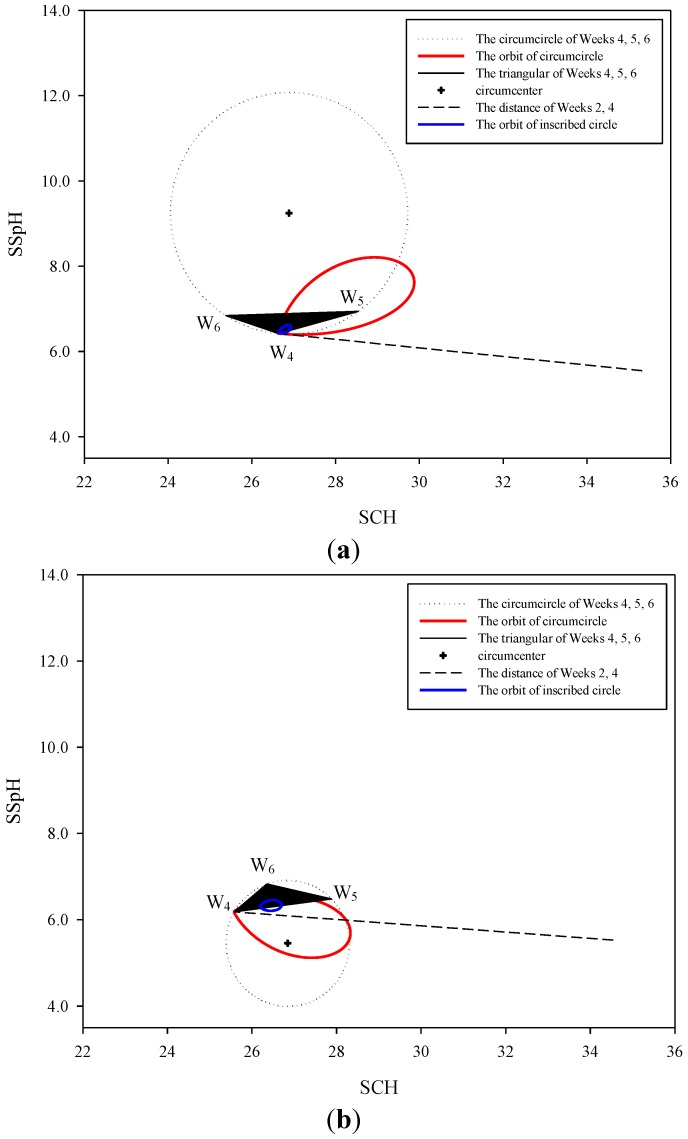
Autonomic nervous system (ANS) performance. (**a**) Left arm. (**b**) Right arm.

We used Microsoft Excel 2010 to calculate the SCH-SSpH values derived from the means at weeks 4 to 6, with triangles forming the incentre and circumcentre (Figure 1). The points on the circumference of the incentre and the SCH-SSpH values obtained at weeks 2 and 4 were used to form numerous triangles. The incentres obtained from the triangles were used to produce one blue spindle-shaped closed curve (Figure 2). In addition, the points on the circumference of the circumcentre and the SCH-SSpH values obtained at weeks 2 and 4 were used to form numerous triangles. The incentres obtained from the triangles were used to produce a red spindle-shaped closed curve (Figure 2).

### 2.5. Signal Operation Diagram Generation

**Figure 3:**
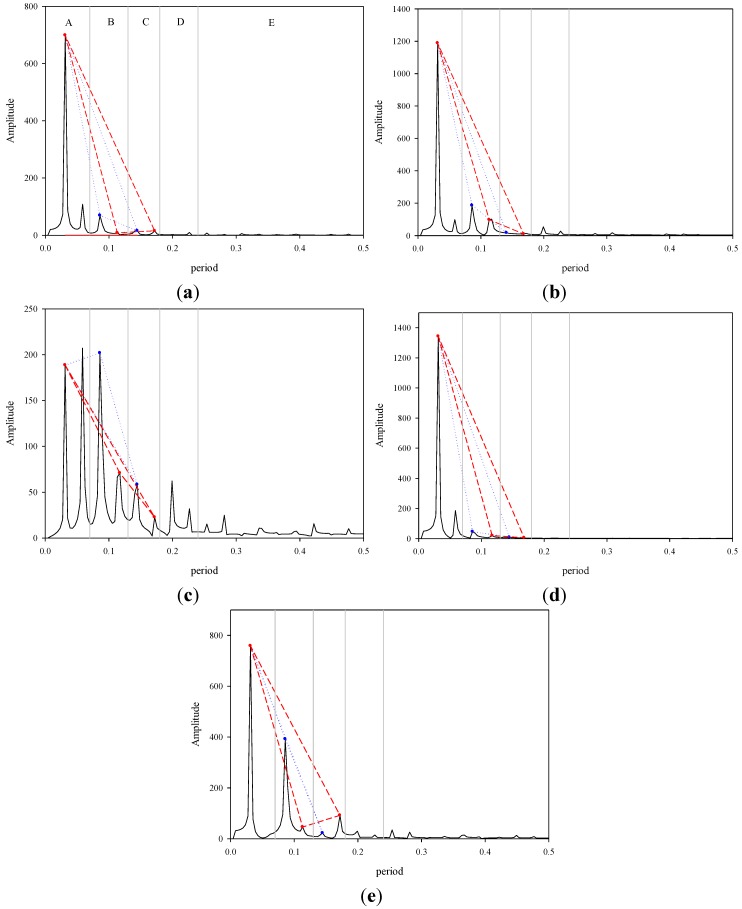
Signal operation diagram. (**a**) Mean value spectrum; (**b**) Case 1; (**c**) Case 2; (**d**) Case 3; (**e**) Case 4.

The points on the circumference of the incentre or circumcentre and the SCH-SSpH values obtained at weeks 2 and 4 were used to form numerous triangles. Each incentre of a triangle has a circumference, and each circumference was pulled in a straight line and arranged side by side to form a wave. Thus, two waveforms for the left arm and two waveforms for the right arm were produced. The two waveforms for the left arm were combined into one waveform. We subsequently used the Fourier formula to transform the waveform (using Microsoft Excel 2010) and obtained a signal operation diagram showing frequency and cycle (Figure 3). The Fourier transform, which is a time-domain and frequency-domain mutual conversion, was performed to obtain the desired information.

## 3. Results and Discussion 

### 3.1. Changes in the Autonomic Nervous System and Orbits Observed in the Natural Environment

The red and blue spindle-shaped closed curves of the triangles derived from weeks 4, 5, and 6 represented the earth’s orbit around the sun and the orbit of the moon, respectively. They also represented the changes in temperature and time associated with SNS and parasympathetic nerve system (PNS) performance (Figure 2). During the 6-week experiment, the SCH-SSpH values obtained at week 2 and week 4 were used to define model formation and the differentiating displays of the model, respectively. The model formation indicated the effect of signals on nerves, which triggered the regulation mechanism, and the differentiating displays of the model illustrated that the nerves that had previously regulated external signals retained the model for regulating subsequent signals. 

The intersection of the red spindle thread origins with the week 4 SCH-SSpH exhibited the highest energy and temperature and the lowest hydration level. At that time, the SNS was highly excited; however, the transmission speed declined, indicating that the excitation responses were concentrated in a few areas of the body. However, an opposite result was observed in the part at which the origin of the red spindle threads extended from week 4 to the vertex. The position of the SCH-SSpH value in Week 5 determined whether the circumscribed circles derived from weeks 4, 5, and 6 overlapped the straight lines derived from the SCH-SSpH values obtained at weeks 2 and 4, thus affecting the overlap ratio of the spindle threads to the straight lines derived from the SCH-SSpH values obtained at Weeks 2 and 4 and that of the spindle threads to the circumscribed circles. The red spindle threads on the right arm almost completely overlapped the circumscribed circles, indicating that the changes in hydration were primarily caused by the changes in temperature. The changes were divided into two sections by the straight lines derived from the SCH-SSpH values in weeks 2 and 4. The intersections of the spindle thread origins and the two sections represent a highly energised, highly excited SNS. This result suggested that, without p-334, the SNS would become excited sporadically. A portion of the spindle threads on the left arm was located outside the circumscribed circles, indicating that p-334 affected the hydration levels in addition to changes in temperature.

The blue spindle threads represented PNS performance. As the two vertices approached the center, the PNS transmission speed decreased, whereas the level of excitation increased. Conversely, the effect of PNS on the level of hydration was relatively low. The spindle threads on the right arm did not fall completely within the triangles, whereas those on the left arm almost completely overlapped the triangles, suggesting that the ANS was not completely involved in the effect of applying skin cream on the skin, but was strengthened by p-334. However, although the blue spindle thread area was larger on the right arm than on the left arm, the circumscribed circles formed by weeks 4, 5, and 6 were smaller, indicating that the ANS-regulation response to skin cream application was inefficient, but was improved by adding p-334.

### 3.2. Signal Operation Diagram

After Fourier transformation of the waves was formed by each circumference, the ANS yielded a clear result regarding regulation of skin signals (Figure 3a). The peaks shown in the chart represent the diurnal variations resulting from the earth’s rotation and indicate the relative relationships between the SNS and the PNS. We used Peaks 1, 3, and 5 to indicate changes that occurred during the day and Peaks 1, 4, and 6 to indicate changes that occurred at night. In addition, the peaks represent the orbit of energy conversion. The triangular area formed by Peaks 1, 3, and 5 (∆135) represents the fixed hydration after metabolism occurred (mean = 16.99), and the area formed by Peaks 1, 4, and 6 represents the amount of water that evaporated (mean = 20.52). The results indicated that the changes in SCH during weeks 4 to 6 were within the difference between the two triangular areas (Figure 1).

Peaks in a pair were defined as a unit, and five units were created, namely, A, 0 to 0.07; B, 0.07 to 0.13; C, 0.13 to 0.18; D, 0.18 to 0.24; and E, 0.24 to 0.50 (Figure 3). The five units represented the neural transmission frequency range, and a decrease in energy level was observed in Regions A to E. Furthermore, the results derived from Regions A to E indicated that p-334 converted luminous energy into other forms of energy. A pair of peaks in Region A indicated that p-334 engaged in energy conversion, suggesting electron transfer and water production. From Regions B to D, the level of energy conversion tended to be minimal. Regions A to D represented the energy performance of the skin, and Region E represented the NS. After sensory signals were received, the signal transmission pathway was receptors → sensory nerves → spinal cord → brain stem → cerebrum → brain stem → spinal cord → motor nerves → effectors (Table 1). A low spacing value indicated high monoamine content and that the body was undergoing repair. Conversely, a high spacing value stimulated the transformation of neural excitation. This region was the fingerprint region of individual participants, and the performance of this region can be used to establish an association between diseases and participants with abnormalities.

### 3.3. Natural Orbits and a Diagram of Signal Operation in the Human Body

From the orbit results (Figure 2), the intersection of the spindle thread origins and the SCH-SSpH value at week 4 marked the time at which the earth was closest to the sun; these results were consistent with the results of a previous study indicating that SNS activity increases in the northern hemisphere during winter [29]. Another study observed elevated heart rates during the new moon and full moon because, during these periods, the SNS was more active that it was during other moon phases [13], indicating a location distant from the two vertices of the blue spindle threads (Figure 2).

Because the magnitude of the change in human body temperature throughout the day differs amongst the various parts of the body [30], we calculated the difference between angles ∠135 and ∠146 in the signal operation diagram (Figure 3a) and determined that the mean difference was ±0.27 °C. This value represented the energy stored in the body during the day. The human body temperature is typically lower during the day than during the night [30] because, during the day, the body undergoes metabolic processes that produce chemicals such as adenosine triphosphate and water, whereas, during the night, the body repairs damaged cells such as brain cells [31], thereby producing heat and causing water to evaporate.

The spacing between the nine peaks in Region E indicated the transmission time for each nerve segment. Only three spacing values, 0.0234 period (p), 0.0273 p, and 0.0313 p, which represented the monoamine content, were observed. Monoamines are associated with the adaptive changes that occur during thermoregulation [32]. The changes in body temperature that occur when skin cream is applied are believed to be related to the changes in monoamine content. Monoamines contain norepinephrine and serotonin, two neurotransmitters that affect neuronal outgrowth [33].

### 3.4. Discussion of the Signal Operation Diagrams of Four Participants

After analyzing the changes in the mean values derived from the participants shown in Region E of the signal operation diagram, we deduced that the transmission time from the receptors to the brain stem was 0.0273 p for all of the participants. Conversely, the transmission time from the brain stem to the motor nerves varied amongst the participants. The transmission time from the motor nerves to the effector was 0.0273 p, indicating that the neurotransmission responses and conversion of excitation from the brain stem to the motor nerves were recurring phenomena (Table 1). Based on the signal operation diagram, we chose to discuss four participants who exhibited abnormalities.

**Table 1:** The meaning of the peak values derived from Region E, Figure 3, the mean values, and the spacing between the peaks.

Receptor		→	Sensory nerve		→	Spinal cord		→	Brain stem		→	Cerebrum		→	Brain stem		→	Spinal cord		→	Motor nerve		→	Effector	
Mean	0.0273			0.0273			0.0273			0.0273			0.0313			0.0234			0.0313			0.0273			
Case 1	0.0273			0.0273			0.0273			0.0313			0.0273			0.0273			0.0273			0.0273			
Case 2	0.0234			0.0273			0.0313			0.0273			0.0273			0.0273			0.0234			0.0273			
Case 3	0.0273			0.0313			0.0234			0.0273			0.0313			0.0273			0.0234			0.0313			
Case 4	0.0234			0.0273			0.0273			0.0273			0.0273			0.0313			0.0273			0.0273			
Case 5	0.0273			0.0313			0.0234			0.0313			0.0234			0.0313			0.0273			0.0273			

After the tests were completed, we asked the participants to describe the neurological illnesses that they had experienced. Participant 1 experienced damage to the optic nerves, which caused a loss of sight. We determined that the signal value from the receptors to the sensory nerves in Region E differed from the mean (Table 1). In Region A of the signal operation diagram, we observed that the first peak was high, indicating that the energy requirement was high. High peaks were still observed in Region D, suggesting that the p-334 in the region still possessed high energy, which should be used to repair cells (Figure 3b). We subsequently calculated the areas of triangles ∆135 and ∆146, which were 22.79 and 26.22, respectively. Regarding water content, a high mean value indicated high levels of energy conversion and water evaporation. The angle difference was 0.02°, which indicated that energy storage was low.

Participant 2 experienced amyotrophic lateral sclerosis (ALS), which is a motor neuron disease [34]. ALS caused Region E to exhibit an abnormality between the motor nerves and the effector (Table 1). The pair of peaks in Region A were nearly identical in height, and the first peak exhibited a low energy demand and p-334 energy conversion rate because the motor nerves consumed relatively little energy (Figure 3c). From Regions B to D, a decreasing trend was normal, and the angle difference was 0.24°, which was close to the mean. However, the areas of triangles ∆135 and ∆146 were 4.31 and 1.14, respectively, indicating that the energy depleted was more than 20% of the mean and that the amount of water evaporated was even lower, and thus signifying that the activity of Participant 2 declined.

ALS is a hereditary disease [34]. Participants 2 and 3 were sisters, and, although no ALS symptoms were observed in Participant 3, an abnormality was observed in Region E (Table 1). Glutamate-induced excitotoxicity is related to the ALS pathology [34], in which p-334 amplifies the effect of ALS. We observed that the SNS of Participant 3 became excited during signal input. The performance of Participant 3 in Region A of the signal operation diagram was similar to that of Participant 1 (Figure 3b,d). The areas of triangles ∆135 and ∆146 were 36.99 and 33.00, respectively, indicating that the participant’s metabolism was high before the incidence of ALS. The angle difference, 0.11°, was lower than the mean value. Furthermore, a distinctive characteristic was observed: the amount of water evaporated was lower than the amount of remaining water.

Participant 4 had no family history of diseases. However, the participant often experienced numbness from the cervical vertebrae to the left palm. When examining Region E, we detected an abnormality from the sensory nerves to the brain stem region (Table 1). The second peak in Region A nearly disappeared, suggesting energy conversion problems. Because the free radicals produced an excess of electrons, the first peak in Region B was extremely large, illustrating the second energy conversion facilitated by p-334. The irregular altitude of the peaks in Regions B to D indicated an energy conversion disorder (Figure 3e). The areas of triangles ∆135 and ∆146 were 0.65 and 22.78, respectively, indicating problems in performing energy metabolism. We calculated that the angle difference was 0.08°, which was lower than the mean; thus, additional body examinations should be conducted in advance.

## 4. Conclusions 

We investigated the regular changes in the skin that occur after skin cream is applied. During weeks 4 to 6, the incentres and circumcentres of the produced orbits indicated PNS and SNS performance, the values of which were similar to those operating in nature. The changes in hydration, temperature, and ANS excitation level followed a fixed orbital pattern. In week 5, in which changes in mode memory were emphasised, we observed that the initial ANS did not fully respond to the signal performance, but was improved by adding p-334. Subsequently, the ANS responded fully and effectively, indicating that performance improved.

We converted the changes in circumference by using the Fourier transformation formula to demonstrate neural signal operation performance, which was divided into five regions. Regions A to D indicated the correlations amongst diurnal energy conversion, water evaporation, and energy fluctuations. Region E was the individual fingerprint region, which enabled us to establish a correlation between the various diseases and abnormal neurotransmissions in the various neural segments. Thus, we developed a monitoring model that can be used to provide precautions for disease outbreaks.
